# Genetically shaping morphology of the filamentous fungus *Aspergillus glaucus* for production of antitumor polyketide aspergiolide A

**DOI:** 10.1186/1475-2859-13-73

**Published:** 2014-05-20

**Authors:** Menghao Cai, Ying Zhang, Wei Hu, Wei Shen, Zhenzhong Yu, Weiqiang Zhou, Tao Jiang, Xiangshan Zhou, Yuanxing Zhang

**Affiliations:** 1 State Key Laboratory of Bioreactor Engineering, East China University of Science and Technology, Shanghai 200237, China

**Keywords:** Filamentous fungi, *Aspergillus glaucus*, Genetically morphology shaping, Shear stress, Aspergiolide A

## Abstract

**Background:**

For filamentous fungi, the basic growth unit of hyphae usually makes it sensitive to shear stress which is generated from mechanical force and dynamic fluid in bioreactor, and it severely decreases microbial productions. The conventional strategies against shear-sensitive conundrum in fungal fermentation usually focus on adapting agitation, impeller type and bioreactor configuration, which brings high cost and tough work in industry. This study aims to genetically shape shear resistant morphology of shear-sensitive filamentous fungus *Aspergillus glaucus* to make it adapt to bioreactor so as to establish an efficient fermentation process.

**Results:**

Hyphal morphology shaping by modifying polarized growth genes of *A. glaucus* was applied to reduce its shear-sensitivity and enhance aspergiolide A production. Degenerate PCR and genome walking were used to obtain polarized growth genes *AgkipA* and *AgteaR*, followed by construction of gene-deficient mutants by homologous integration of double crossover. Deletion of both genes caused meandering hyphae, for which, Δ*AgkipA* led to small but intense curves comparing with Δ*AgteaR* by morphology analysis. The germination of a second germ tube from conidiospore of the mutants became random while colony growth and development almost maintained the same. Morphology of Δ*AgkipA* and Δ*AgteaR* mutants turned to be compact pellet and loose clump in liquid culture, respectively. The curved hyphae of both mutants showed no remarkably resistant to glass bead grinding comparing with the wild type strain. However, they generated greatly different broth rheology which further caused growth and metabolism variations in bioreactor fermentations. By forming pellets, the Δ*AgkipA* mutant created a tank environment with low-viscosity, low shear stress and high dissolved oxygen tension, leading to high production of aspergiolide A (121.7 ± 2.3 mg/L), which was 82.2% higher than the wild type.

**Conclusions:**

A new strategy for shaping fungal morphology by modifying polarized growth genes was applied in submerged fermentation in bioreactor. This work provides useful information of shaping fungal morphology for submerged fermentation by genetically modification, which could be valuable for morphology improvement of industrial filamentous fungi.

## Background

Filamentous fungi are morphologically complex microorganisms producing a variety of useful products containing proteins and secondary metabolites. The use of fungi for the production of commercial products has increased rapidly over the past decades [1]. As is known, filamentous fungi grow in the form of dispersed mycelia, clumps and pellets in submerged culture [2]. The basic growth unit of hyphae usually makes it sensitive to shear stress generated from mechanical force and dynamic fluid. It then severely affected the outputs of microbial products, which remains as a persistent hardness in bioreactor fermentations. Besides, the more dispersed the mycelia are, the easier they are damaged by shear force.

The conventional strategies against this problem always focus on adapting agitation, impeller type, bioreactor configuration and type [3-6]. In bioreactor culture, agitation is necessary to fully mix the gas-phase and liquid-phase and promote mass transfer, facilitating the homogeneous distribution of the medium components, pH, temperature, and dissolved oxygen tension (DOT). Low agitation speed would negatively affect fermentation process despite of mycelia protection. However, strong agitation would initiate high shear stress which causes damage to mycelia and affects metabolite production, especially for the shear-sensitive filamentous fungi [1,7,8]. Besides, although the impeller modifying and bioreactor remodeling could facilitate shear-sensitive fermentation to some extent, the high cost and tough works always raised many problems in bioreactor fermentations especially for the large-scale industrial applications.

*Then what if engineering hereditable fungal morphology so that the fungi could be adapted to the most commonly used bioreactor and the fermentation could be easily and successfully scaled up for industrial application?* A brilliant work has performed by BASF company (Germany) that they screened a mutant of filamentous fungus *Beauueria bassiana* LU700 with special yeast-like morphology by natural mutation with UV and chemicals. The resulted (R)-2-(4-hydroxyphenoxy) propionic acid productivity finally increased by 23.3 folds in bioreactor [9]. However, as we know, the process of natural mutation is quite random and time-consuming. Thus, with the rapid fungal molecular biology development, rationally shaping morphology by genetic modification turns to be preference.

In recent years, the molecular biological study on the polarized growth of filamentous fungi was developed widely [10-14]. In *Aspergillus nidulans*, hyphal extension is oriented according to the environmental conditions and depends on a continuous flow of vesicles to the growing hyphal tip. The vesicles contain various enzymes and factors essential for cell wall and membrane extension and accumulate as the Spitzenkörper. Besides, microtubules (MT), actin, motor proteins, cell end factors or landmark proteins are involved in establishment and maintenance of polarity [10]. Deficient of either kinesin motor KipA or cell end marker TeaR caused curved hyphae due to the disordered polarized growth [15,16]. Thus, these innovative achievements might be applied to directionally shape the inheritable fungal morphology for establishment of an efficient bioprocess.

*Aspergillus glaucus* HB1-19 is a marine-derived filamentous fungus. Among its numerous secondary metabolites, a novel polyketide [17], aspergiolide A, markedly inhibited proliferation of cancer cell lines [18]. Recently, the animal test showed that aspergiolide A could inhibit tumor growth effectively in model mice (unpublished data). For further pharmacology and pharmacodynamics studies, a large amount of aspergiolide A is necessary. However, *A. glaucus* HB1-19 is very sensitive to shear stress, and adding two glass beads (diameter 5 mm) before inoculation in shake flask culture decreased aspergiolide A production by 89.1% and more beads caused even worse results [19]. In bioreactor fermentation, aspergiolide A production only reached 35.0% of that in shake flask even after optimizations of oxygen provision and impellers combination [19]. Thus, it met with many difficulties in bioreactor culture and further fermentation scale-up.

This study aims to engineer shear resistant morphology for aspergiolide A production of shear-sensitive marine-derived *A. glaucus* HB1-19 by modifying its polarized growth genes. By this work, we could also evaluate the practicability of the idea that genetically shaping shear-resistant morphology for improving submerged fermentation of filamentous fungi.

## Results

### Gene cloning, analysis and deficient strain construction

The sequences of ORF and flanking regions of *AgkipA* and *AgteaR* were obtained by degenerate PCR and nested PCR, followed by reverse transcription to identify the amino acid sequences. The *AgkipA* ORF is 2909 bp with an intron at 782–861 bp site, which encoded 946 amino acids (GenBank accession No. KC822644). The *AgteaR* ORF is 1686 bp with an intron at 103–159 bp site, which encoded 542 amino acids (GenBank accession No. KC822645). AgKipA exhibits high identity with the homologs in other *Aspergillus* spp*.* (*A. clavatus* 74%, *A. fumigatus* 71%, *A. nidulans* 70%, *A. niger* 77%, *A. oryzae* 72%, *A. terreus* 74%, Additional file 1: Figure S1A). AgTeaR also exhibits good identity with the homologs in other *Aspergillus* spp. (*A. clavatus* 52%, *A. fumigatus* 51%, *A. nidulans* 49%, *A. niger* 51%, *A. oryzae* 53%, *A. terreus* 52%, Additional file 1: Figure S1B)*.* The conserved protein domain analysis by SMART (http://smart.embl-heidelberg.de/) indicates that AgKipA owns a KISc domain (Kinesin motor, catalytic domain. ATPase) at 214–576 aa site, and AgTeaR harbors a CAAX prenylation motif at the C terminus similarly to the *S. pombe* Mod5 and *A. nidulans* TeaR [16,20]. The phylogenetic trees of AgKipA and AgTeaR were shown in Additional file 2: Figure S2, indicating the close relationship to those of other *Aspergillus* spp*.*. DNA linear fragments of the deletion cassette pKS10/pRS20 were transformed into the wild type (WT) *A. glaucus* HB 1–19 and the positive transformants were then confirmed by PCR analysis and further DNA sequencing.

### Comparison of Δ*AgkipA*, Δ*AgteaR* and WT strains in solid culture

The phenotypes greatly changed after deletion of *AgkipA* or *AgteaR* (Additional file 3: Figure S3). Colony of Δ*AgkipA* and Δ*AgteaR* mutants resembled pretty much the WT strain after cultivation on MM agar plate for 4 d. For both mutants, polarity defect was also detected in the way the second germ tube emerged from conidiospore. In the WT strain, the second hypha emerged from the side of the spore opposite to the germ tube (bipolar) in 81% of the spores. In contrast, the bipolar behavior only happened in 23% of the spores in the Δ*AgkipA* mutant and 20% of the spores in the Δ*AgteaR* mutant, respectively. Development of *A. glaucus* HB 1–19 was also investigated. *A. glaucus* reproduces sexually in homothallic type [21]. In this work, the mutants and WT strain produced similar numbers of sexual cleistothecium and asexual conidiospore, which indicated that *AgkipA* and *AgteaR* exercised little influence on the development of *A. glaucus* HB 1–19.

**Figure 1:**
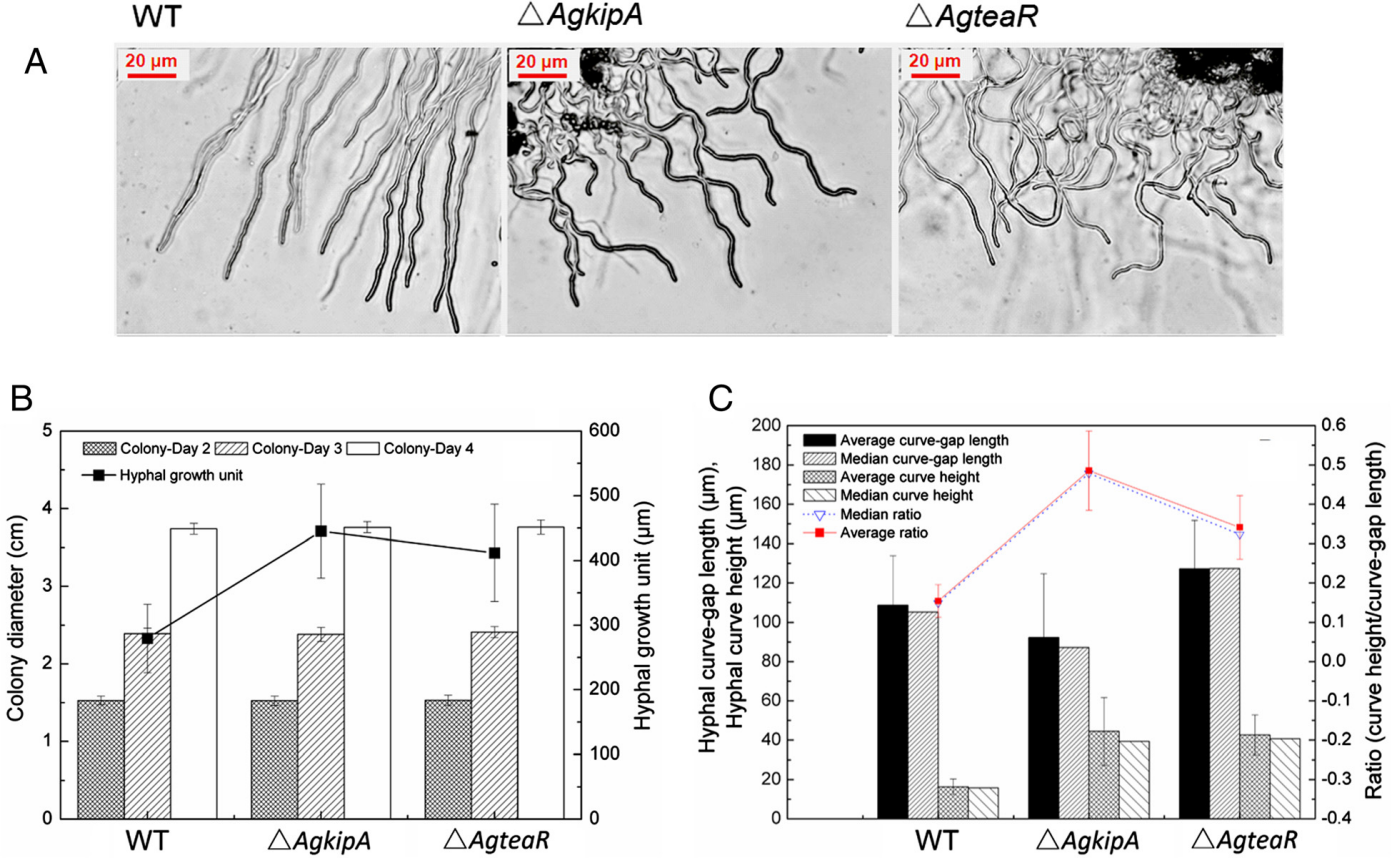
**Colony and hyphal growth of Δ*Agkip*A, Δ*AgteaR *and wild type strains. (A)** Hyphae (400 ×) of Δ*AgkipA*, Δ*AgteaR* and wild type strains observed by a Leica DM3000 microscope. Spores were inoculated on coverslips with 0.5 ml MM agar plates and grown at 30°C for 20 h; **(B)** Colony growth and hyphal growth unit; **(C)** Hyphal curve evaluation. The parameters referred to those marked in Figure 1. For each experiment, 50–60 objects were analyzed for every strain. The measurement methods were described in the Section of Analytical methods.

**Figure 2:**
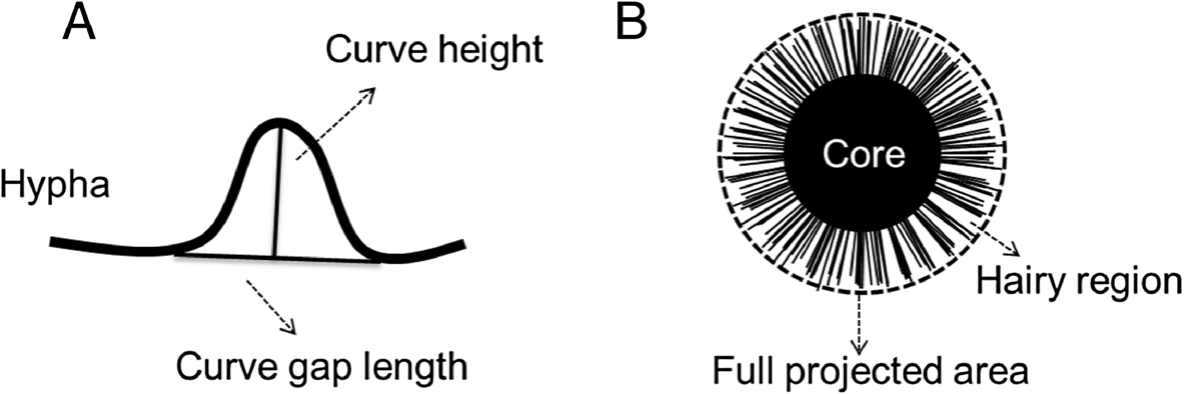
**Schematic drawing of hyphal curve (A) and mycelial morphology (B).** The ratio (hyphal curve height/hyphal curve gap length) was used to evaluate the hyphal curvature. The ratio (core projected area/full projected area) was applied for analysis of fungal morphology.

What is interesting and important is, deletion of *AgkipA* and *AgteaR* caused meandering hyphae (Figure 1A). To analyze the differences between both types, the hyphae was then quantified. Deficiency of *AgkipA* and *AgteaR* did not affect colony radial growth, while they improved the hyphal growth unit (HGU) (Figure 1B). The Δ*AgkipA* mutant showed a HGU of 445.1 μm at 36 h, which was 59.4% and 8.1% higher than the WT and Δ*AgteaR* mutant, respectively. However, hyphal diameter resembled each other among three strains (about 6.0 μm). To quantify hyphal curvature, defined parameters including hyphal curve height, hyphal curve gap length and their ratio were involved in analysis (Figure 2). All three parameters differed significantly among the three strains (*P value* <0.01) and the results were shown in Figure 1C. The Δ*AgkipA* mutant presented the maximal curve height and minimal curve gap length, leading to the highest ratio (0.49 ± 0.1) of hyphal curve height/curve gap length, which was confirmed as the most curve hyphae. For Δ*AgteaR* and WT, the ratios of hyphal curve height/curve gap length were 0.34 ± 0.08 and 0.15 ± 0.04, respectively. It proved that hyphae of the Δ*AgteaR* mutant were much more curve than the WT strain. Moreover, the Δ*AgteaR* mutant possessed the maximal hyphal curve gap length but a relatively high hyphal curve height, meaning that its hyphal curves were very large. The WT strain had few curves which always showed the minimal hyphal curve height but large hyphal curve gap length, indicating that these curves were quite mild. The median for each parameter was also presented and median close to average value further confirmed the statistical validity of the elements in analysis.

### Comparison of Δ*AgkipA*, Δ*AgteaR* and WT strains in shake flask culture

**Figure 3:**
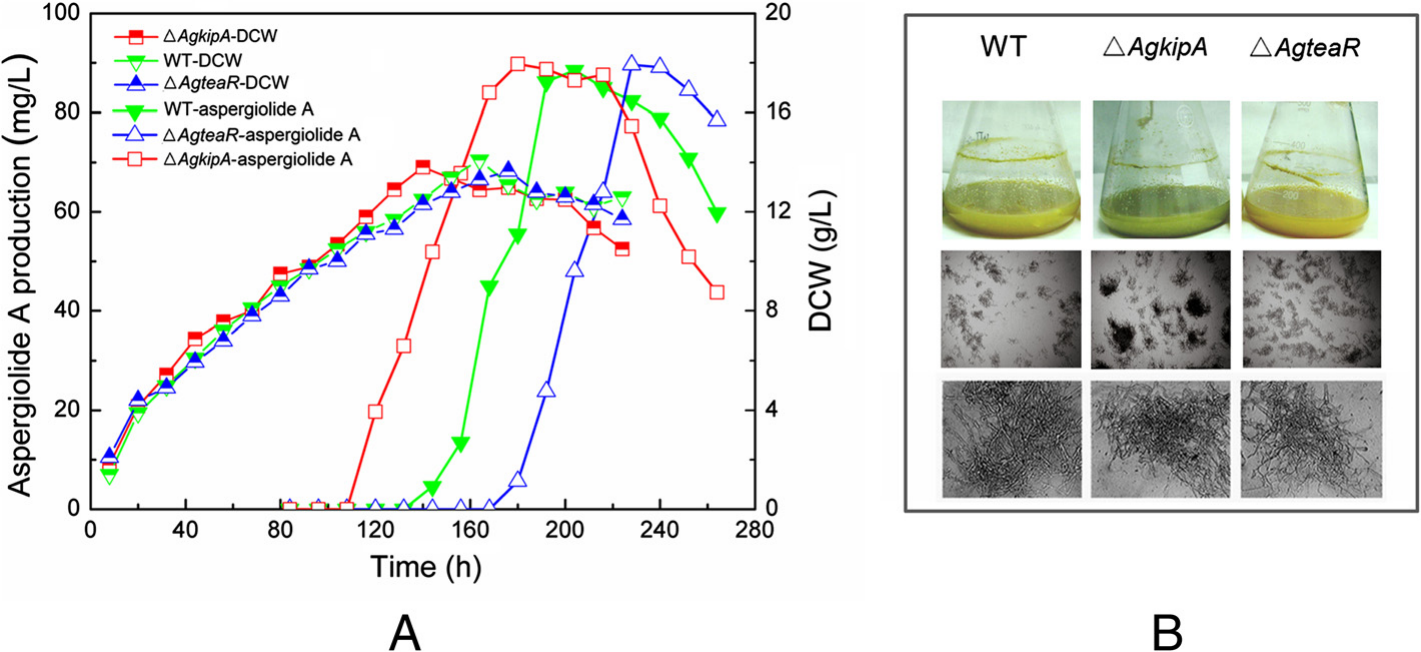
**Time course profiles of aspergiolide A production and morphology of different strains in shake flask culture. (A)** Dry cell weight and aspergiolide A production; **(B)** Fungal morphology. Upper, broth color; Middle, morphology (40×); Bottom, morphology (100×). The photos only reflected the morphology but not biomass concentration.

The three strains were then fermented in shake flask to evaluate production variations of aspergiolide A caused by deletion of polarized growth genes. As shown in Figure 3A, the highest aspergiolide A production and dry cell weight (DCW) were almost the same among all three strains. However, the time for production vertex in the Δ*AgkipA* mutant was advanced by 24 h compared to the WT strain (204 h), whereas that in the Δ*AgteaR* mutant was delayed by 24 h. The similar phenomenon also showed up in biomass accumulation, meaning that aspergiolide A production was closely related with mycelial growth. The color of the broth is extremely related to secondary metabolism in *A. glaucus* HB1-19. As shown in Figure 3B, the WT produced dark-yellow broth, whereas the broth of the Δ*AgkipA* mutant showed greenish-yellow and that of the Δ*AgteaR* mutant presented golden-yellow after 3 d culture. Commonly, when the culture going on, the broth color of *A. glaucus* HB1-19 turned dark until the final dark brown. Simultaneously, aspergiolide A production also experienced an increasing process. Therefore, in this case, the different broth colors of *A. glaucus* strains conformed with the time courses of aspergiolide A productions (Figure 3A). Interestingly, the genetically engineered curved hyphae also organized different clumps from the WT strain in liquid culture (Figure 3B). Comparing to the WT strain, the Δ*AgkipA* mutant preferred to form more compact clumps while the Δ*AgteaR* mutant used to generate a bit looser clumps.

### Evaluation of shear resistance of Δ*AgkipA,* Δ*AgteaR* and WT strains and their fermentations in bioreactor

As previously reported, bead grinding and impeller agitation could destroy mycelia of *A. glaucus* and negatively affected aspergiolide A biosynthesis [19]. Therefore, to analyze the shear resistance and process diversities of the genetically shaped morphology of both mutants, fermentations were performed involving mechanical shear stress resulted from bead grinding in shake flask or impeller agitation in bioreactor.

**Figure 4:**
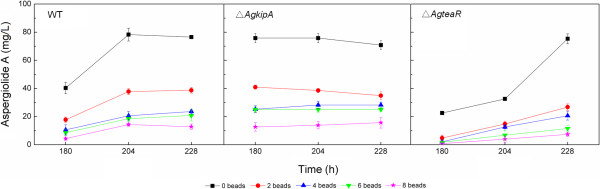
**Effects of different number of glass beads on aspergiolide A production by different strains.** Three time points (108, 204 and 228 h) were considered. Glass bead diameter was 5 mm. Glass beads were added before inoculation.

Figure 4 shows aspergiolide A production of the WT, Δ*AgkipA* and Δ*AgteaR* strains after cultivation for 180, 204 and 228 h, respectively, in shake flasks added with different numbers of beads. The results indicated that beads (diameter of 5 mm) severely damaged either the WT strain or the mutants. As a result of bead grinding, the mycelia became loose, short and fragile. Addition of two glass beads reduced aspergiolide A production by 51.8%, 70.5% and 53.4% in the Δ*AgkipA*, Δ*AgteaR* and WT strains, respectively. Increasing bead number further decreased the production. Therefore, neither Δ*AgkipA* nor Δ*AgteaR* mutant showed remarkable resistance to bead grinding as compared to the WT strain.

**Figure 5:**
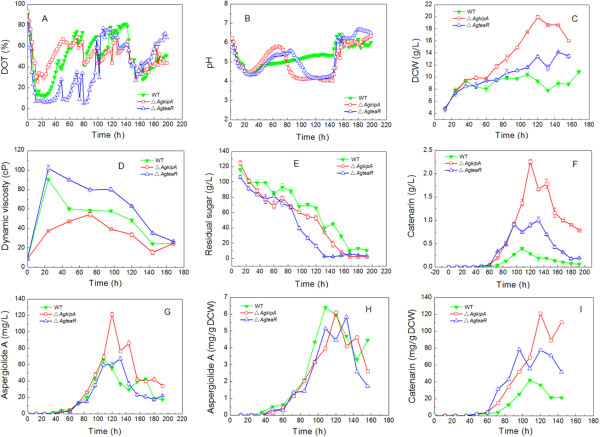
**Time profiles of the *A. glaucus *strains of wild type, Δ*AgkipA*and Δ*AgteaR *in 5-L bioreactor fermentations. (A)** Dissolved oxygen tension (DOT); **(B)** pH variations; **(C)**, Dry cell weight (DCW); **(D)** Dynamic viscosity; **(E)** Residual sugar; **(F)** Catenarin production; **(G)** Aspergiolide A production; **(H)** Aspergiolide A productivity; **(I)** Catenarin productivity.

In 5-L bioreactor fermentation, the three strains showed apparently different traits in spite of the same inoculation and operation conditions (aeration, agitation, temperature, inner tank pressure, etc.). As shown in Figure 5A, DOT decreased quickly during the early growth phase for all the three strains. However, in Δ*AgkipA* culture DOT kept at a higher level than the WT strain, while Δ*AgteaR* was opposite. The pH variations also changed a lot in the gene-deficient strains. Both Δ*AgkipA* and Δ*AgteaR* mutants showed an obvious process of pH going down and keeping constant during 68–144 h, which absolutely differed from the WT strain (Figure 5B). The Δ*AgkipA* mutant produced the highest DCW of 19.9 ± 0.3 g/L (120 h), which was 48.5% (144 h) and 89.5% (108 h) higher than the Δ*AgteaR* mutant and the WT strain, respectively (Figure 5C). For sugar consumption, Δ*AgteaR* mutant showed the fastest utilization, which seemed not completely in accordance with DCW curves (Figure 5C, E). The highest dynamic viscosity of Δ*AgteaR* mutant reached 101.8 ± 3.0 cP, which was 12.7% and 86.8% higher than that of the WT strain and Δ*AgkipA* mutant, respectively (Figure 5D). The time point (72 h) of the highest dynamic viscosity of Δ*AgkipA* mutant delayed for 48 h comparing with other two (Figure 5D). Difference of broth color was similar to that in shake flask culture (Figure 3B), and broth color of Δ*AgkipA* mutant became deep much faster than others. The Δ*AgkipA* mutant generated the highest production of aspergiolide A (121.7 ± 2.3 mg/L), which was 82.2% and 80.3% higher than the WT strain and Δ*AgkipA* mutant, respectively (Figure 5F). The important intermediate of aspergiolide A, catenarin, also showed the highest production (2.3 ± 0.1 g/L) in the Δ*AgkipA* mutant, which was 475% and 130% higher than that in the WT strain and Δ*AgteaR* mutant, respectively (Figure 5G). For productivity of aspergiolide A, the WT strain (6.39 mg/g DCW) showed the highest value but it was only 5% and 9.6% higher than the Δ*AgkipA* and Δ*AgteaR* mutants, respectively. However, for productivity of catenarin, the Δ*AgkipA* mutant (121.2 mg/g DCW) was 189.6% and 56.6% higher than the WT strain and Δ*AgteaR* mutant, respectively (Figure 5H, I). Different from shake flask culture, the time point for production vertex greatly shortened for all three strains. Moreover, in bioreactor the WT strain reached the highest productions of both compounds at 108 h, which were 12 and 24 h, respectively, earlier than those of Δ*AgkipA* and Δ*AgteaR* mutants (Figures 3A & 5F, G).

**Figure 6:**
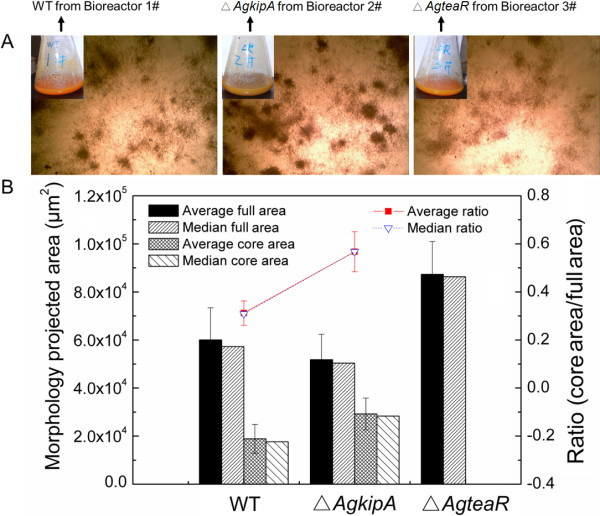
**Fungal morphologies of different strains in 5-L bioreactor fermentations. (A)** Morphologies photos (40×) of the wild type, Δ*AgkipA* and Δ*AgteaR* strains from 24 h culture; **(B)** Quantification of the morphologies. The parameters referred to those marked in Figure 2.

The distinctions of broth viscosity must be correlated with fungal morphology. As shown in Figure 6A, the Δ*AgkipA* mutant formed compact pellets even in 24 h culture. Conversely, Δ*AgteaR* always generated loose clumps. The WT strain produced the modest morphology comparing to both mutants. To quantify the morphology, a 48 h culture broth was selected for analysis. The Δ*AgkipA* mutant and WT strain turned to form pellet with a compact core and an accessory hairy region, while the Δ*AgteaR* mutant mostly produced relatively loose clump without core region. It clearly showed that the Δ*AgkipA* mutant easily formed bigger cores but with smaller hairy region, which then caused a smaller full mean projected area ((5.18 ± 1.06) × 10^4^ μm^2^) of pellet and a higher ratio of core area/full area (0.57 ± 0.08), as compared to the WT strain (Figure 6B).

For *A. glaucus* HB1-19 fermentation, the viscosity of broth increased with mycelia growing and biomass accumulation. The broth showed similar characteristics to Newtonian fluids under certain condition (see Methods), thus we simplified it as Newtonian broth for comparative analysis. For Newtonian fluid, the shear stress can be denoted by the equation τ = μγ (τ, shear stress; μ, dynamic viscosity; γ, shear rate of local region in bioreactor). Thus the volumetric averaged shear stress in different domains in bioreactor can be calculated. Here, the volumetric averaged shear stress was calculated by computational fluid dynamics (CFD) modeling at the time point when the broth dynamic viscosity reached the maximal level of 54.5, 101.8 and 90.3 cP (Figure 5D), respectively, for the Δ*AgkipA* mutant, Δ*AgteaR* mutant and WT strain. The mesh diagram of impeller for inner and outer fluid domain of 5 L tank was shown in Additional file 4: Figure S4A. The calculated results stated that the volumetric averaged shear stress in up impeller zone, down impeller zone and tank zone of Δ*AgkipA* mutant are all the lowest while those of Δ*AgteaR* mutant are all the highest (Additional file 4: Figure S4B).

## Discussion

In bioreactor fermentation, mycelial growth of filamentous fungi usually faces great physical damage from growing environment. Thereinto, shear stress created by impeller agitation often breaks the hyphae violently especially the shear-sensitive strains. Moreover, fungal morphology may change broth rheological properties and mass transfer capabilities, which then vary cell metabolism and culture process [1]. The marine-derived *A. glaucus* HB 1–19 is such a shear-sensitive strain bringing troubles in fermentation scale-up [19,22]. In this work, we make an effort to genetically shape its morphology to resist to shear stress and improve broth rheological properties with the purpose of enhancing aspergiolide A production.

The fungus showed no colony growth delay or significant development variation by disrupting polarized growth genes *AgkipA* and *AgteaR*. However, both gene deficient mutants showed defect in hyphal orientational growth and conidiospore bipolar germination. Thus both AgKipA and AgTeaR are necessarily involved in its polarized growth. Previous research in *A. nidulans* also indicated that KipA and TeaR determined growth directionality, interacting with other structures such as landmark protein TeaA and TeaC, formin protein SepA, microtubules and actin cytoskeletons, *etc*. [15,16,23]. Deletion of *AgkipA* and *AgteaR* made high HGU value, indicating that the hyphae of both mutants became longer than the WT strain. The Δ*AgkipA* mutant produced highly curved hyphae and formed solid pellets in submerged culture. However, the Δ*AgteaR* mutant generated hyphae with large curve but with mild curvature and formed clumps looser than the WT strain. This could be attributed to that, the highly meandering hyphae with many small intense curves made them easily and intensely bind togeother while the hyphae with large mild curves made them crisscross and support mutually to leave more space inside the clumps.

Although the curved hyphae should be more flexible theoretically, they showed no remarkable shear resistance to bead grinding as compared to the wild type. It might contribute to that the strong and insistent grind from bead-mycelia-bead and bead-mycelia-flask inwall which severely destroy the mycelia. However, Δ*AgkipA* mutant formed typical pellets, which led to low-viscous broth, low shear stress, high DOT and high biomass accumulation in bioreactor fermentation. Consequently, Δ*AgkipA* mutant produced the highest production of aspergiolide A and its intermediate catenarin. It is curious that Δ*AgteaR* mutant showed different characteristics of morphology, growth and metabolism both in shake flask and bioreactor fermentations. A possible reason might be that, Δ*AgteaR* grew well in the beginning but the long hyphae with mild curves could not effectively form pellet. It then increased broth viscosity and decreased oxygen transfer, which made it hardly form compact pellet further and slowed down cell growth [1]. The similar aspergiolide A productivity and greatly different catenarin productivity indicated that catenarin formation was not the limited step in the biosynthesis of final product. Enhancing biomass growth and increasing the intermediate which reacts with catenarin to form aspergiolide A would be important for aspergiolide A biosynthesis.

The Δ*AgkipA* mutant grew faster than the WT strain and Δ*AgteaR* mutant in bioreactor, which could be caused by a lower viscosity and better oxygen supply in broth of the pelleted Δ*AgkipA*. For all three strains, aspergiolide A was produced earlier in bioreactor fermentation than that in the shake flask. It might be related to the good aeration and mixing in bioreactor. Our previous study on impeller combination and oxygen carrier feeding showed that a good dissolved oxygen and mixing condition would shorten fermentation phase [19]. Also, the Δ*AgkipA* mutant reached the production vertex 12 h later in bioreactor but 24 h earlier in shake flask as compared to the WT strain. A possible reason is that impeller somewhat loosed the pellets and made it more suitable to grow so that the secondary metabolism delayed.

Morphology control for filamentous fungus is often a prerequisite for industrial application, especially the shear-sensitive fungus. Many chemical and physical factors, *e.g.*, medium, inoculum, dissolved oxygen, impeller type and agitation, *etc.*, could affect fungal morphology [1]. However, morphology shaped by these factors was not genetically stable. Moreover, the optimized factor for desired morphology might destroy metabolic balance for the objective product. In this work, the morphology of shear-sensitive *A. glaucus* HB 1–19 was rationally shaped by genetic modification on hyphal polarized growth genes and the application in bioreactor fermentation worked well. A gorgeous result was obtained that aspergiolide A production from the Δ*AgkipA* mutant reached 121.7 ± 2.3 mg/L, which increased 82.2% comparing with the WT strain. During the late phase of the culture, aspergiolide A production from the Δ*AgkipA* mutant decreased quickly, which showed similar tendency to the WT and Δ*AgteaR* strains (Figure 5G). This might ascribe to the intrinsic cell metabolism. This work contributed more to production enhancement of aspergiolide A in contrast with the former bioprocess and bioengineering optimizations. It illuminates that gene engineering acts as a powerful tool in morphology shaping for fermentation of filamentous fungus, which could also be used in fermentation of industrial fungi such as citric acid producing *A. niger*, lovastatin producing *A. terreus*, protease-producing *A. oryzae*, penicillin producing *Penicillium spp.*, pigment producing *Monascus* spp., and so on.

## Methods

### Strains and culture conditions

*Escherichia coli* Top10 (Invitrogen) was used as a host for construction of plasmids and was grown at 37°C in LB medium (supplementing 2% agar and/or 50 μg/ml ampicillin when needed). *A. glaucus* HB1-19 (CCTCC M 206022), provided by Ocean University of China [18], was used as a recipient strain for construction of Δ*AgkipA* and Δ*AgteaR* mutants. Medium for conidiation, preculture and fermentation were previously described [19,24]. After cultivation in conidiation medium at 30°C for 5 d, about 10^8^ spores were inoculated to 100 ml preculture medium and incubated at 28°C for 16 h with shaking at 180 rpm to produce the fresh mycelia for protoplast preparation and genomic DNA extraction. For screening gene deletion mutants, MEA medium composed of 2% malt extract, 2% glucose, 0.1% peptone, 2% agar dissolved in distilled water was used with addition of 100 μg/ml Zeocin. Sorbitol (1 M) was added to the MEA medium in order to maintain the osmotic pressure balance inside and outside the cytomembrane. Hyphae were observed by growing on minimal medium (MM) [25].

### Molecular techniques

Degenerate PCR and nested PCR was applied to obtain the target gene sequences. A genome walking kit (TaKaRa, cat. no. 6108) was used to clone the full gene sequences. To determine the intron-extron borders, cDNAs was amplified by reverse transcription-PCR [26] using primescript II 1st strand cDNA synthesis kit (TaKaRa, cat. no. D6110S). *A. glaucus* HB1-19 was transformed by protoplast transformation [27]. DNA sequencing was performed commercially (Shanghai Sangon Biotech Co. Ltd, China). Genomic DNA was extracted using plant genomic DNA kit (Tiangen Biotech Co., Ltd., China). For PCR experiments, standard protocols were applied following PCR amplification kit (TaKaRa, cat. no. R011).

### Deletion of *AgkipA* and *AgteaR*

Δ*AgkipA* and Δ*AgteaR* mutants were constructed by homologous integration of double crossover with the Zeocin resistance gene *Sh ble* as a marker. The primers used are listed in Table 1. The resistance gene fragment P*trpC*-*Sh ble*-T*trpC* was amplified by PCR from pUC-LZ with primers SF and SR [27]. The 2.2-kb fragment was digested with *Kpn*I and *Bam*HI and inserted into the plasmid pUC18 (Invitrogen), yielding pUC18-*Sh ble*. The 1.6-kb upstream and 2.1-kb downstream regions of *AgkipA* flanked by the ORF were amplified by PCR using genomic DNA as template and KUF/KUR and KDF/KDR as primers, then digested with *Eco*RI/*Kpn*I and *Bam*HI/*Sph*I and cloned into the pUC18-*Sh ble*, yielding pKS10. DNA linear fragment amplified with primers KUF/KDR was transformed into *A. glaucus* HB1-19. The positive transformants were confirmed by PCR analysis and DNA sequencing. The *AgteaR* deletion plasmid, named as pRS20, was constructed in the same way except for the amplification of 2-kb upstream and 2.2-kb downstream fragments using primers RUF/RUR and RDF/RDR, respectively.

**Table 1:** List of primers used in this work

**Primer**	**Sequence(5’-3’)**
SF	CGGGGTACCATGCCAGTTGTTCCAGTGATCTTCG
SR	CGCGGATCCCTCGAGTGGAGATGTGGAGTGGGC
KUF	CGGAATTCGAACGCAGTTGGTTAGTCTGGGACA
KUR	GGGGTACCAGGCCATGGTGATCTGGTCGATAA
KDF	CGGGATCCCGATCACTGCGTTCTTCCGTCAGTC
KDR	ACATGCATGCATGGAATCATCTCTCATGGCGCG
RUF	CGGAATTCCCCGCGTATGCGATG
RUR	CGGGATCCATGAGGTCAGACCAGCTGAACCCGT
RDF	CGGGATCCGCTCCATTGGCTATTGCTACCGACG
RDR	ACATGCATGCGTGCAGTTACCGAAGGCGCTGG

### Fermentation in shake flask and bioreactor

For shake flask culture, 10^8^ spores were inoculated into 100 ml seed medium and incubated at 28°C for 48 h with shaking at 180 rpm to produce the inoculum. Afterwards, 10 ml broth was processed with an air pump filtration and washed with 1 L distilled water. The biomass was dried at 100°C for 1 h and measured dry cell weight (DCW, g/L). Then, for all three strains, inoculums with different volumes equal to the same dry biomass (0.032 g DCW) were inoculated into 250 ml Erlenmeyer flask containing 50 ml fermentation medium and incubated at 28°C with shaking at 180 rpm for 8–9 d. Different numbers of glass beads (diameter of 5 mm) were added to shake flask before inoculation to evaluate shear resistance of Δ*AgkipA*, Δ*AgteaR* and WT strains [19].

For fermentation in bioreactor, the inoculum was prepared as same as described above. Then inoculums with different volumes equal to the same dry biomass (1.932 g) were transferred into 3.0 L fermentation medium in three same 5 L stirred tank bioreactors (Shanghai Guoqiang Bioengineering Equipment Co., Ltd, China). The working volume was 3.5 L, and the equipped impeller was double layer six-blade Rushton disc turbine (RDT, 6.8 cm i.d.). The lower impeller was 2.5 cm above the reactor bottom, and the vertical distance between two impellers was 7.2 cm. The aeration system was an air inlet through a ring sparger with air-flow meter. A jacketed water bath was designed to maintain process temperature. Dissolved oxygen tension (DOT) was measured using a polarographic probe (Oxyferm FDA, Hamilton, Switzerland) calibrated to 100% saturation for aeration of 1 vvm in the medium at agitation of 600 rpm and tank inside pressure of 0.02 Mpa. For all batches, the fermentation temperature was kept at 28°C. The agitation speed was controlled at 300 rpm (0–14 h), 375 rpm (14–80 h), 350 rpm (80–100 h) and 300 rpm (100 h to the end), respectively. The aeration was adjusted to 1.0 vvm (0–10 h), 1.67 vvm (10–62 h), 1.38 vvm (62–120 h), 1.0 vvm (120–144 h) and 0.2 vvm (144 h to the end), respectively.

### Analytical methods

Observation and count of conidiospore and cleistothecium as well as measurement of colony growth were described previously [28]. Hyphal growth unit (=hyphal length/number of apex) were assessed under light microscopy equipped with a CMOS camera and an image analytical software package TSView (XSP-BM-8CA, BM Optical Instruments Manufactory, Shanghai, China) after 36 h culture on MM medium evenly coating at a microscope slide. Spore germination was observed by the same light microscopy and 200 objects were analyzed for each strain. Hyphal curvature was evaluated by a curve parameter (curve height/curve gap length) using the same microscopy system and culture method (Figure 2A). A high value of curve parameter means a high degree of hyphal curve. Pellet morphology analysis was modified from the previous studies [29,30]. Fungal pellets usually consist of a central compact core region and a peripheral filamentous or hairy region [29,30]. Thus, the morphology has been characterized in terms of a pellet core projected area and full projected area (core + hairy region) (Figure 2B) after 48 h bioreactor fermentation in this study. A high value of core area/ full area indicates a compact pellet. For each experiment, 50–60 objects were analyzed for determination. Median and *P value* were used to estimate the statistical validity and significance. Analysis of residual sugar and aspergiolide A production was as same as before [28]. Catenarin was analyzed referring to the previous study [31]. Liquid viscosity analysis was performed on 15 ml broth samples collected from the bioreactor at regular intervals by a programmable digital viscometer (DV-2 + Pro, Shanghai Nirun Intelligent Technology Co., Ltd., China) with rotor and adapter for small amount sample controlled at 200 rpm, and 28°C (accessary water circulation thermostat). The bioreactor geometry was incorporated into the commercial CFD software CFX 14.0 (ANSYS Inc., Canonsburg, PA) to calculate the shear stress. The fluid was simplified as the uniform liquid phase with the same viscosity value. The multiple reference frame (MRF) method was used to model the steady state flow and the value of convergency criteria was set to 10^-4^ for double layers of six-blade Rushton disc turbine (RDT) impeller. The viscosity of *A. glaucus* fermentation broths turned to be constant and its power law index (n) became nearly 1.0 under viscometer rotor rotating at above 110 rpm, it has similar characteristics to Newtonian fluids under intense rotation conditions. As the agitation of impeller was always controlled at above 300 rpm, we simplified it as Newtonian fluids for comparative analysis and used single-phase flow Newtonian model in CFX to simulate and evaluate the averaged shear stress in different domains (Additional file 4: Figure S4) in bioreactor fermentation.

## Competing interests

The authors declare that they have no competing interests.

## Authors’ contributions

MC designed and conducted the fermentation experiments and morphology quantification. YZ constructed the strains and conducted phenotype analysis. WH performed part of phenotype analysis and morphology photographing. MC and YZ analyzed the results and wrote the manuscript. XZ and YZ reviewed the manuscript. All other authors participated in this work. All authors have read and approved the final manuscript.

## Supplementary Material

Additional file 1: Figure S1 Alignment of KipA **(A)** and TeaR **(B)** homologues from different *Aspergillus* species. Click here for file

Additional file 2: Figure S2 Phylogenetic trees of KipA **(A)** and TeaR **(B)**. Click here for file

Additional file 3: Figure S3 Phenotypic comparison of the Δ*AgkipA*, Δ*AgteaR* and wild type strains. Click here for file

Additional file 4: Figure S4 Shear stress in the fermentation in 5-L bioreactor that simulated by computational fluid dynamics. Click here for file
