# Supplementary material for: Genetically shaping morphology of the filamentous fungus Aspergillus glaucus for production of antitumor polyketide aspergiolide A
Source: Microb Cell Fact. 2014 May 20;13:73. doi: 10.1186/1475-2859-13-73 (PMC4039328; doi:10.1186/1475-2859-13-73)
Supplement: Additional file 1: Figure S1 — Alignment of KipA (A) and TeaR (B) homologues from different Aspergillus species. [file 1475-2859-13-73-S1.docx]

A


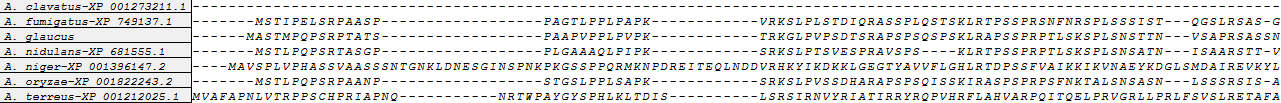


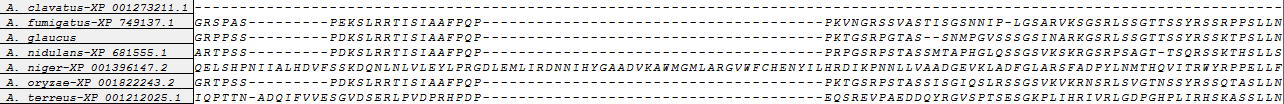


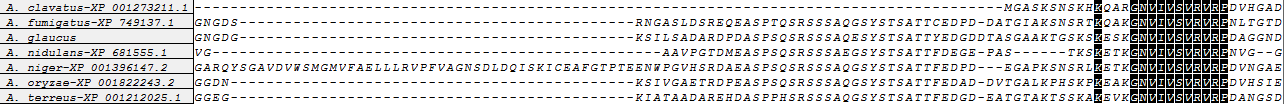


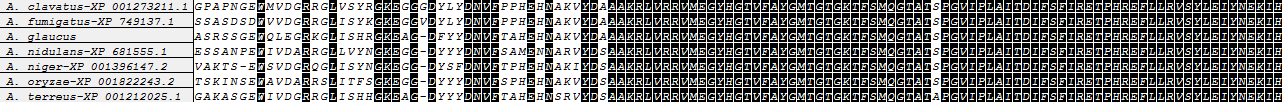


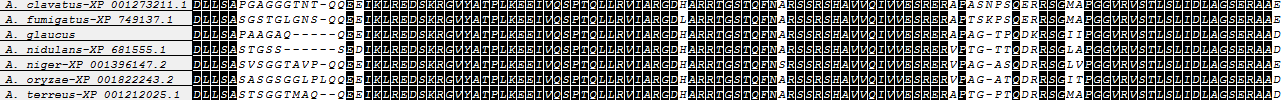


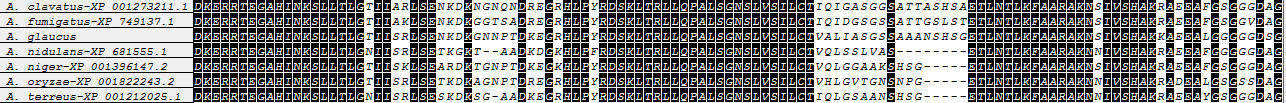


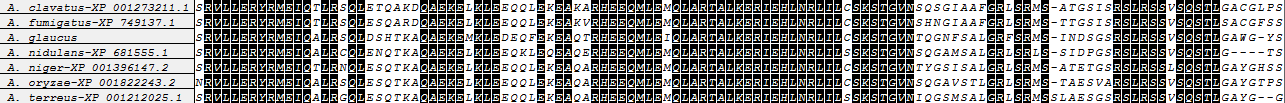


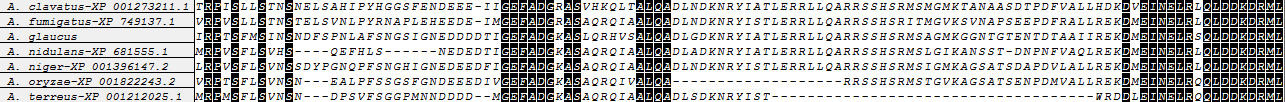


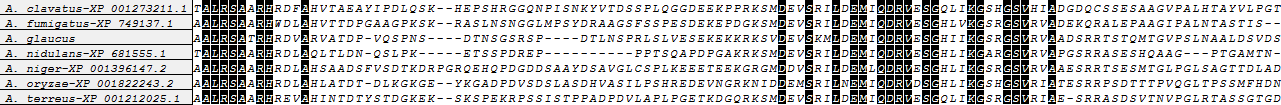


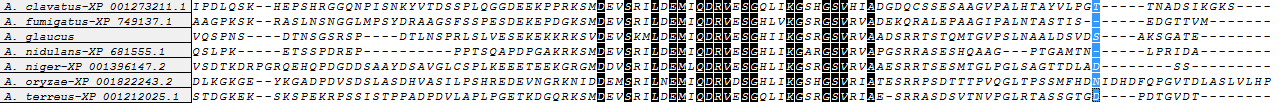

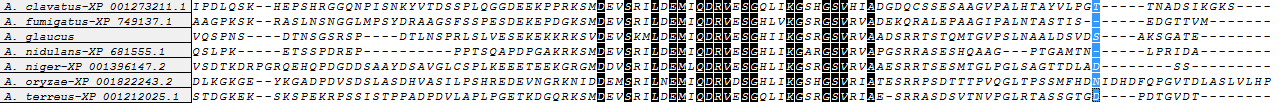


B


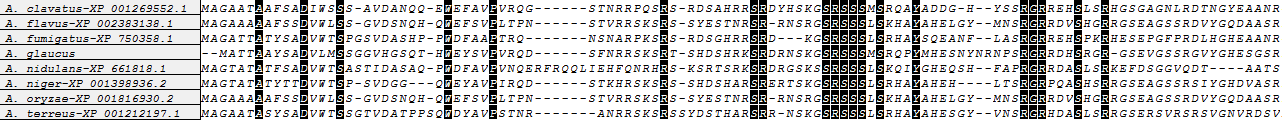


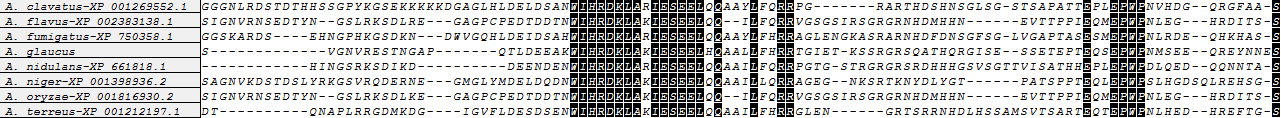


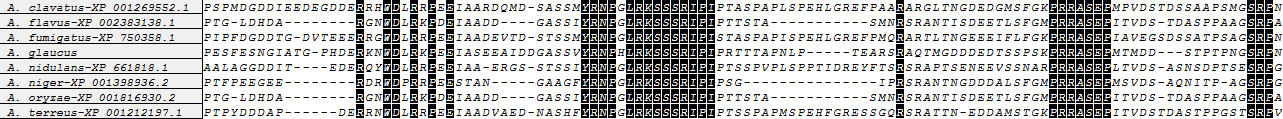


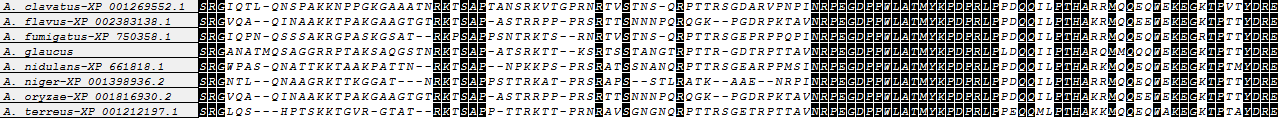


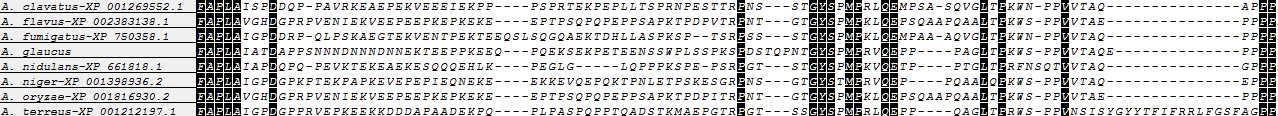


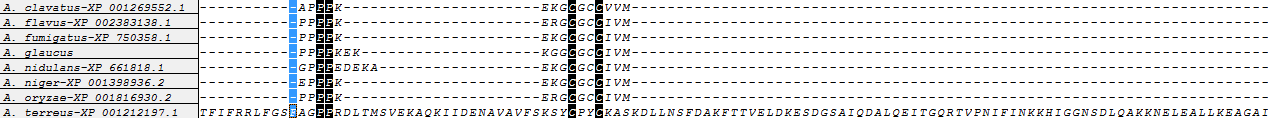

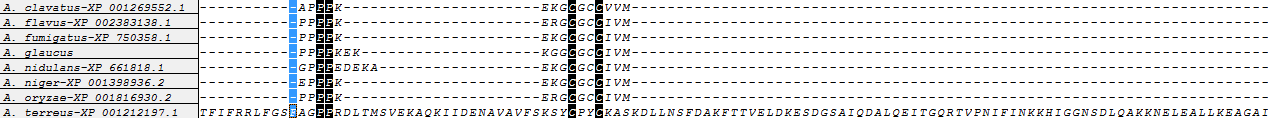


**Suppl. Figure 1** Alignment of KipA (A) and TeaR (B) homologues from different *Aspergillus* species. *A.* *glaucus* KipA (AgKipA) and TeaR (AgTeaR) was obtained and determined in this work. The alignments were done with MEGA 5.05 with standard parameters. Conserved sites at 100% level are shaded in black.
