# Supplementary material for: Genetically shaping morphology of the filamentous fungus Aspergillus glaucus for production of antitumor polyketide aspergiolide A
Source: Microb Cell Fact. 2014 May 20;13:73. doi: 10.1186/1475-2859-13-73 (PMC4039328; doi:10.1186/1475-2859-13-73)
Supplement: Additional file 2: Figure S2 — Phylogenetic trees of KipA (A) and TeaR (B). [file 1475-2859-13-73-S2.docx]

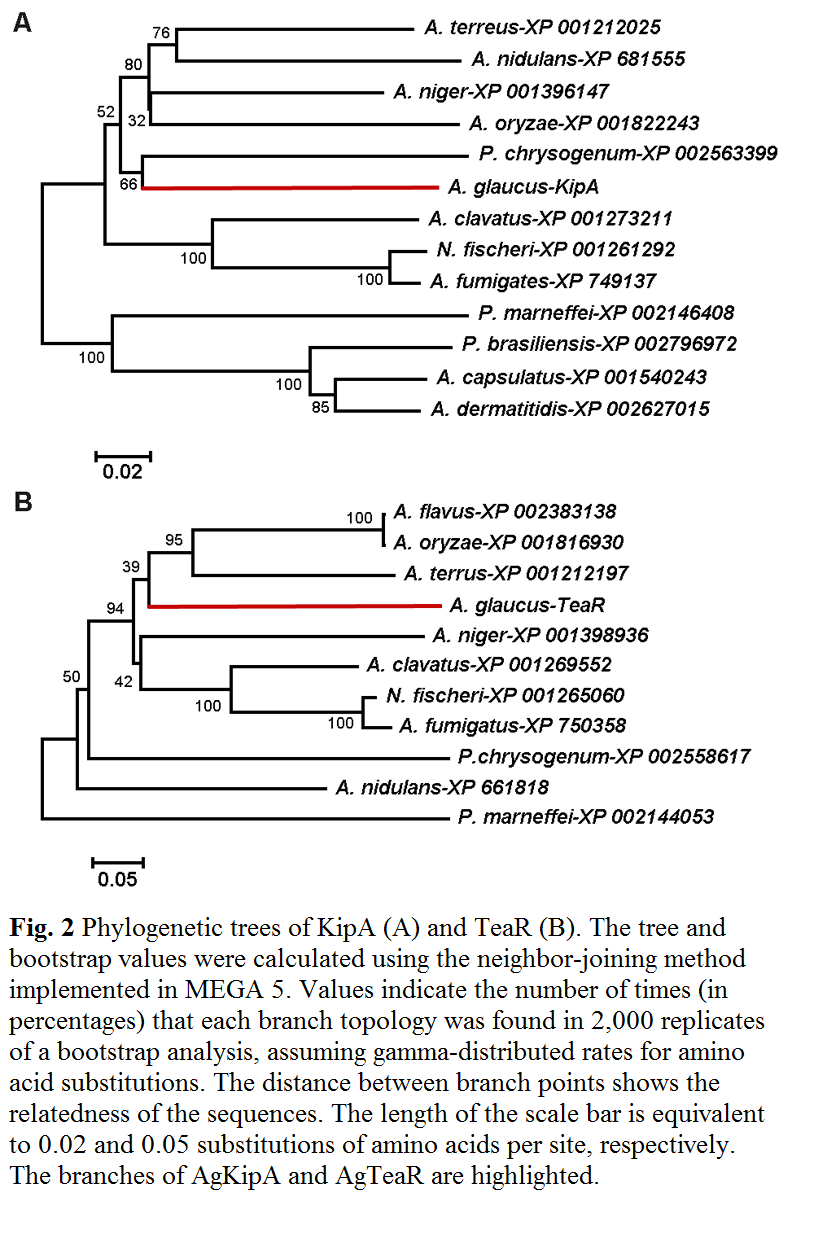


**Suppl. Figure 2** Phylogenetic trees of KipA (A) and TeaR (B). The tree and bootstrap values were calculated using the neighbor-joining method implemented in MEGA 5. Values indicate the number of times (in percentages) that each branch topology was found in 2,000 replicates of a bootstrap analysis, assuming gamma-distributed rates for amino acid substitutions. The distance between branch points shows the relatedness of the sequences. The length of the scale bar is equivalent to 0.02 and 0.05 substitutions of amino acids per site, respectively. The branches of AgKipA and AgTeaR are highlighted.
