# Supplementary material for: Genetically shaping morphology of the filamentous fungus Aspergillus glaucus for production of antitumor polyketide aspergiolide A
Source: Microb Cell Fact. 2014 May 20;13:73. doi: 10.1186/1475-2859-13-73 (PMC4039328; doi:10.1186/1475-2859-13-73)
Supplement: Additional file 3: Figure S3 — Phenotypic comparison of the ΔAgkipA, ΔAgteaR and wild type strains. [file 1475-2859-13-73-S3.docx]

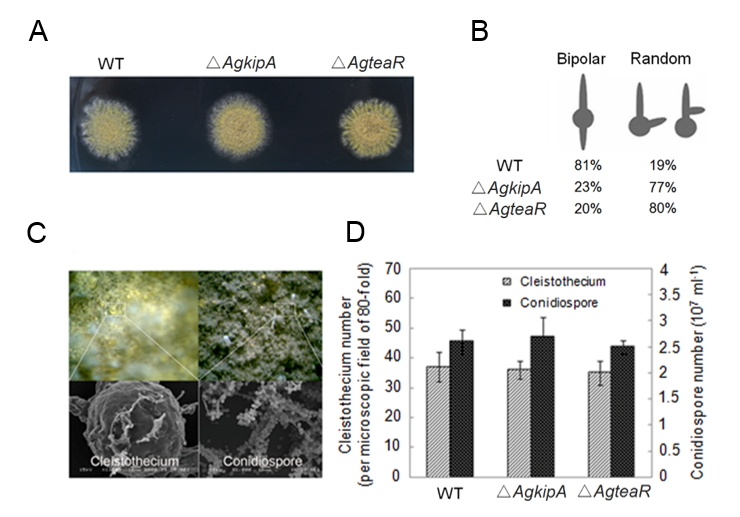


**Suppl. Figure 3** Phenotypic comparison of Δ*AgkipA*, Δ*AgteaR* and wild type strains. (A) Colonies of wild type (WT), Δ*AgkipA* and Δ*AgteaR* strains. Equivalent spores (1.0 × 10^5^) were grown on MM agar plates at 30°C for 4 d. (B) Quantification of the effect of the gene deletions on second germ tube formation. Conidia of each strain were germinated in MM agar plates and then analyzed for the emergence of the second germ tube. For each strain, 200 germlings were counted. (C) Appearances of conidiospore and cleistothecium under stereomicroscope (XTZ-E, BM Optical Instruments Manufactory, Shanghai, China) (80 ×, upper panel of C) and scanning electron microscopic (JSM-6360LV, JEOL, Japan) (1000 ×, down panel of C). This figure was adapted by Cai et al., 2010. (D) Comparison of numbers of conidiospore and cleistothecium of Δ*AgkipA*, Δ*AgteaR* and wild type strains. The measurement methods were described in the Section of Analytical methods.
