# Supplementary material for: Genetically shaping morphology of the filamentous fungus Aspergillus glaucus for production of antitumor polyketide aspergiolide A
Source: Microb Cell Fact. 2014 May 20;13:73. doi: 10.1186/1475-2859-13-73 (PMC4039328; doi:10.1186/1475-2859-13-73)
Supplement: Additional file 4: Figure S4 — Shear stress in the fermentation in 5-L bioreactor that simulated by computational fluid dynamics. [file 1475-2859-13-73-S4.docx]

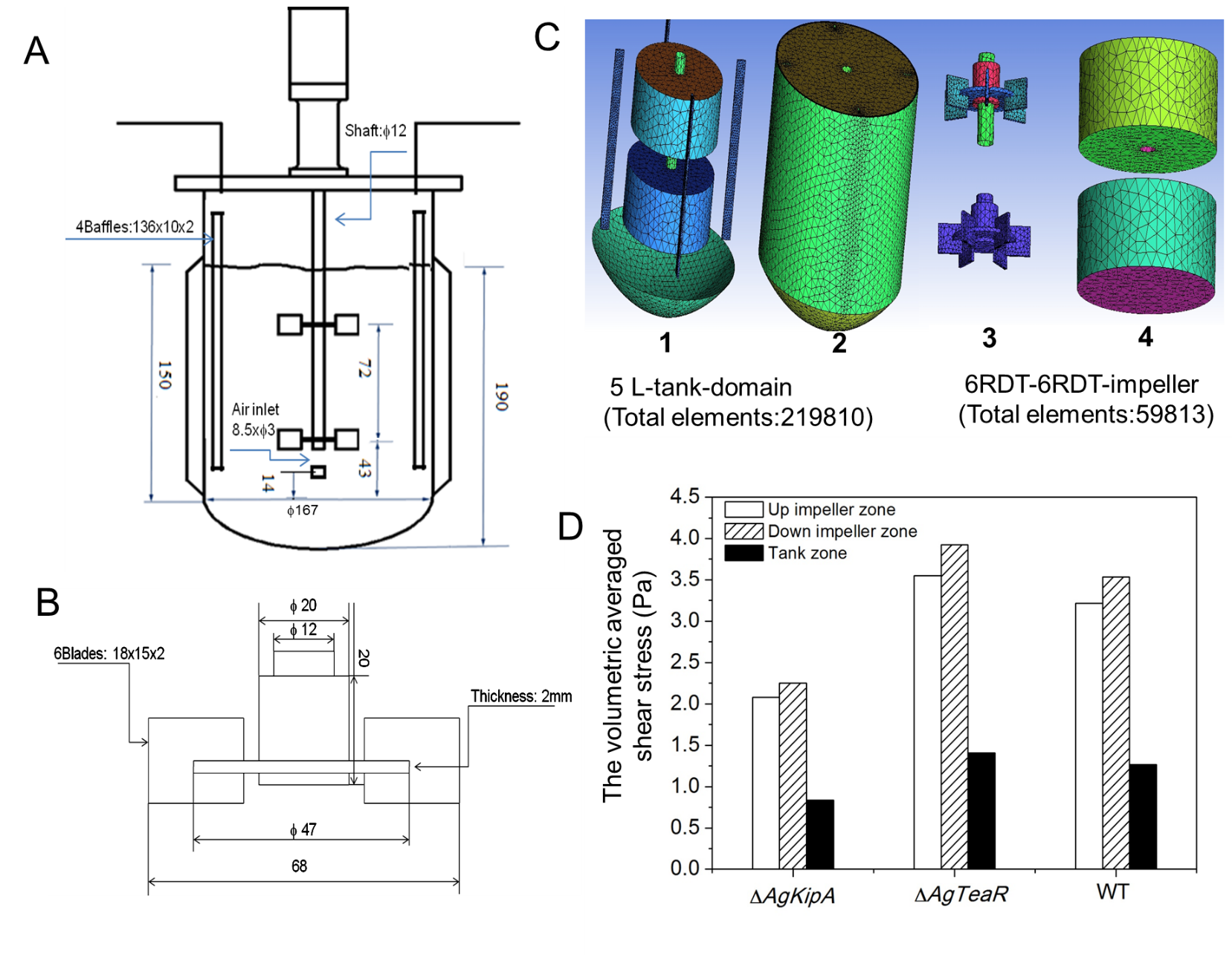


**Suppl. Figure 4** Shear stress in the fermentation in 5-L bioreactor that simulated by computational fluid dynamics. (A) Geometrical parameters of the tank of 5 L bioreactor (Size units: mm); (B) Geometrical parameters of the six-blade Rushton disc turbine (6-RDT) impeller (Size units: mm); (C) The mesh diagram for the inner and outer fluid domain of 5-L tank. C1, surface mesh of interface and baffle; C2, outer tank fluid domain (static region); C3, surface mesh of boundary of impeller and rotation zone; C4, outer impeller fluid domains (rotation zone, for each domain, height 58 mm, radius 53.73 mm). The static region was divided into 219810 elements and the rotation zone was divided into 59813 elements. (D) Shear stress distribution in the bioreactor tank. The multiple reference frame (MRF) method was used to model the steady state flow and the value of convergency criterion was set to 10^-4^. The viscosity of *A. glaucus* fermentation broths turned to be constant and its power law index (n) became nearly 1.0 under viscometer rotor rotating at above 110 rpm, thus it has similar characteristics to Newtonian fluids under intense rotation conditions. The agitation of impeller always controlled higher than 300 rpm. Therefore, it was simplified as Newtonian fluids for comparative analysis and used single-phase flow Newtonian model in CFX to simulate and evaluate the shear stress. The dynamic viscosity of 54.5 cP, 101.8 cP, and 90.3 cP for Δ*AgkipA*, Δ*AgteaR* and WT was involved in calculation, respectively.
